# New Elements of Operative Surgery

**Published:** 1848-01

**Authors:** 


Art. VI.—*New Elements of Operative Surgery.* By Alf. A. L. M. Velpeau, Professor of Surgical Clinique of the Faculty of Medicine of Paris, Surgeon of the Hospital La Charitè, Member of the Royal Academy of Medicine, of the Institute, &c. Carefully revised, entirely remodelled, and augmented with a Treatise on Minor Surgery. Illustrated by over 200 Engravings, incorporated with the text. accompanied with an Atlas in quarto of twenty-two plates, representing the principal operative processes, surgical instruments, &c. First American, from the last Paris edition. Translated by P. S. Townsend, M.D., late Physician to the Seamen's Retreat, Staten Island, New York. Augmented by the addition of several hundred pages of entirely new matter, comprising all the latest improvements and discoveries in Surgery, in America and Europe, up to the present time. Under the supervision of, and with notes and observations by, Valentine Mott, M.D., Professor of the Operations of Surgery with Surgical and Pathological Anatomy, in the University of New York. Foreign Associate of the Acadèmie Royale de Mèdecine of Paris, of that of Berlin, Brussels, Athens, &c. In three volumes. Vol. I. pp. 914: 1845. Vol. II. pp. 1092: 1846. Vol. III. pp. 1162: 1847. New York: Samuel S. & William Wood.

[Second Notice—See Western Journal for July, 1846—vol. vi. p. 50, Second series.]

This translation, a short notice of which we gave on the appearance of the second volume, is at length completed—indeed the last volume has been on our table for two or three months. For ourselves we are greatly rejoiced at it. The tiptoe of expectancy on which we had been kept for many, many months was becoming *too* painful. It is a relief to be once more flat-footed—a source of *positive* happiness not to us only, but to most people "at least in America, if not in other parts of the world." Thank heaven! the unaccustomed sounds, worse than the discord of a Hindoo funeral, wherewith each volume was announced, are now stilled—sounds which not the 'thunderstorm of battles' on our southern border (wherever that may be), not the loud wail of starving Ireland, not the wild cry of Italy clamorous for her freedom, could subdue. Above all these they rose, like

——————'sounds of air
Which had the power all spirits of compelling.'

Heaven be praised, say we, that this clamor, bad as "devils' yule," is subsiding; and with it the myriad-flight of winged advertisements, fanning the air from east to west, and clouding the bright sun. In the partial calm that has succeeded, the publishers' voice is borne to us, requesting a notice, and we intend to comply. The press has groaned under these volumes; compositors, stereotypists, proof-readers, and binders, have groaned over them; and it shall not be our fault if the translator and his "sponsor" do not groan for them.

As each volume appeared, the translator has crowed like chanticleer, has thrown his defiance boldly to the world. He has asserted *iterum iterumque*, and he still asserts, that this translation (with its particular object, end, and aim, its final cause, the glorification of M. Mott) has all excellencies in a superlative degree; and this he will maintain against all adversaries whatsoever. It was not our wish to enter the lists with such a doughty champion, such a 'parlous' and puissant combatant. We felt as though a single *Townsendian* sentence—viz: winding in and out, and interlooped, like thread in a silken purse or a yarn stocking, or twisted around like a *Convolvulus* or a bale of rope—would be the death of us. Therefore it is that we have waited thus some months, hoping and trusting that a stouter hand, a more stalwart arm, an older and a better soldier, would offer himself for this stern and fearsome encounter. But we have waited in vain. Our valorous knight and true, mounted on his gallant Gallic Rosinante, with his faithful squire hard by (knight and squire having for the nonce changed places) still paces back and forth in the arena, 'grace in his step, and fire in his eye!' Three several times has the herald sent forth his loud summons; but yet no answering shout! and now there comes to us the 'still small voice' of the publishers! and now, even as David,

> ————————'armed with simple sling
> And some smooth stones up-gathered from the brook,'

went forth to meet Goliah, so go we forth to meet Townsend. Thus, then, we put our trumpet to our lips—there goes our clarion! and if its notes are now heard faint and indistinct, they shall swell louder and clearer as we proceed.

Before we address ourselves to the work in general, we have a word or two to say touching this "third and last" in particular. We call our readers to witness, that in our previous notice we made an attempt to save this volume (and through it the two former) from its friends, that we endeavored to keep it from inevitable damnation, to 'pluck it as a brand from the burning.' But so strong is it in original sins that it is greatly to be feared our efforts were unavailing. In manner it is precisely like the two former—only 'more so.' It is indeed an exaggerated copy of them. It was pre-signified by the same mighty portents; its approach was announced by similar trumpet-blowings and drum-beatings; it was ushered forth with rather more than the usual amount of solemn satisfaction and swelling self-complacency. In it Townsend, who is the very pink of translators, has reached the 'highest point of all his greatness.' The three volumes in their excellence rise above each other 'as hills o'er hills, and Alps o'er Alps arise.' The first was good, the second better, but this is good in the superlative degree—it is the best. The vocabulary of good names is exhausted upon it. Had there been a fourth, we cannot conceive what the translator could have said, or how he could have found words

(and he is as copious of words as Niagara is of water) to carry him up to 'the height of that great argument.'

It was right then to take unusual pains to recommend so much excellence. Accordingly, besides the customary advertisements, such as confectioners are wont to employ to set forth their wonderful collection of cakes, candies, and comfits, we had the eye addressed in another mode. An idea having been borrowed from 'illustrated Saturday Couriers' and 'pictorial Brother Jonathans,' we had advertisements accompanied with xylographic illustrations; but whether these were addressed to the sense of beauty or that of the marvellous, is not quite clear to the uninitiated. Perhaps to both; but be this as it may, the preliminary praisings and puffings of this third volume were backed up and underborne by a picture of the celebrated Nassau *Nose.* Of the picture (now before us) we may say what Maginn said of a portrait of Rogers, the poet—it is 'a mortal likeness painted to the very death!' As to the nose depicted, it is a real '*monstrum horrendum*, *informe*, *ingens*,' to which the Strasburg nose of Slawkenbergius and Sterne was a mere wart. It is almost as large as the great tower of Strasburg. The Nassau stranger got not only one of the 'goodliest and jolliest' noses from the 'Promontory of Noses,' but he got the very biggest. This wonderful nose (whose history, as learned by us since the reception of the third volume, we may as well relate here) had a much more powerful influence and a much wider sphere of action than Diego's nose. The latter, if our memory serves us, set the sentinel and the bandy-legged drummer, the trumpeter and the trumpeter's wife, to quarrelling; it caused the abbess of Quedlingberg and the four great dignitaries of her chapter—the prioress, deaness, sub-chantress, and senior-canoness—to lie awake three whole nights; it put the faculty by the ears; in fine, it kept all Strasburg 'macerated with expectation' for the space of seven-and-twenty days. But John Russell's nose, even without the accompaniment of 'crimson-satin breeches and a fringed cod-piece,' did all this, and a great deal more, to a much greater number. It kept all Nassau and all the Bahamas and some of the West Indies rocking with anxiety as with an earthquake for a whole score of years! Our translator, happy man! more fortunate than the bandy-legged drummer, or the trumpeter's wife, or the innkeeper's wife, or the burgomaster's widow, got to touch it! ay, marry, did he! and under his plastic hand (by what he calls a movement *en dèdolant*) it became 'by St. Radagunda, a most noble nose!' rather undefined in its shape, but lying somewhere between a mitigated aquiline and a moderated pug. And thus were Nassau and its islanders saved after being tortured through a whole quarter of a century! The Strasburgers, led away by their nose, lost their city; the islanders lost their nose (thanks to Townsend!) and saved their city.

It will be recollected that in the former volumes our translator proved himself a thorough '*annexationist*.' To his philosophic eye, surgery had then quite supplanted medicine in the latter's peculiar province; or, to use his own beautiful and eloquent language, "the great and controlling domain of Practical, Operative, and Pathological and Anatomical Surgery," was then "monopolizing almost the entire healing art." It seems that this monopoly is now complete. Physic is merged in surgery—the greater is sunk in the less. There shall not be even an *imperium in imperio.* Over this broad domain Mott and Velpeau, the Royal *Gemini*, the Siamese twins of surgery, are to reign, with Townsend as their chief mandarin, or prime minister, and their head dragoman. Our translator, as 'lieutenant general' of the forces in this career of conquest (each volume being a separate invading column), will not listen to treaty proposals; he will admit no 'parallel of 37°;' he will not be contented with the 'Upper California' and 'New Mexico' of medicine; he will not set apart a 'Nueces region' for a 'neutral territory;' he will not 'fall back on Gen. Taylor's line,' or 'Mr. Calhoun's line;' but he has just seized and insists on retaining the entire territory—the 'whole of Mexico.' That which we foresaw *in posse*, we now perceive *in esse;* that which was in progress a year and a half a go, is now a 'fixed fact.' We felt certain that to 'this complexion it would come at last;' that our translator's appetite would grow more *bulimious* by indulgence. Like Alexander, he may now weep, for there are no more worlds to conquer. The 'area' of surgery is now enlarged to the utmost limit of capacity—the three dimensions of extension are satisfied—even destiny, 'manifest destiny' (for such it is) cannot further enlarge it. It is not without regret we record this fact; not on our own account so much as on account of some of our friends. Through instinct, or the gift of second-sight, or early prescience of some such catastrophe, *our* leaning has always been towards surgery, and therefore we are prepared for such a step. But among our numerous readers, there are many who—not gifted with our second-sight—not having our "prophetic souls"—not forewarned and therefore not forearmed—have not had such leaning; or, if they have, they have been condemned like our translator to "investigate febrile diseases as personally examined and investigated" by themselves. To such this wholesale annexation will be unwelcome news, it will be at least for a time calamitous. Let all such come to this goodly city and its flourishing school, which is destined to be the great centre of Townsend's surgical 'area.' In the mean time, let us all be of good cheer. As old Nautes said to Eneas when the Trojan women in a frolic had burned nearly all his ships:

> 'Quicquid erit, superanda omnis fortuna ferendo est.'

This sudden reverse of fortune merely exemplifies the axiom that 'power is always stealing from the many to the few.' We can dwell on the past glories of medicine—we can live again in the memory of the past. We can look at the blue and red stripes of the barber's pole, and recall that time when *we*, the doctors, lorded it over the surgeons; when surgeons were barbers and barbers were surgeons; when they smoothed rough chins and plastered sore shins, anointed the head and bled, prepared pomatums and mixed medicamentums, paired nails and pulled teeth, dressed bag-wigs and set broken legs; in short, when, besides their tonsorial offices, they *did* all sorts of surgical operations "under the immediate supervision and by the advice" of us the doctors. Then they were our menials, our bondmen, our slaves; and though the tables are now turned, we may look to the future with large faith and hope, knowing that 'the whirlgig of time will bring in its revenges.'

But releasing this third volume from our mordacious grasp for a moment, let us place it along side of the others, and take a look at the "*ensemble.*" There they stand, ranged like a rampart around us, constituting our visible bound whereby we are 'cabined, cribbed, confined.' They are our apparent and true horizon, above which the "atlas of two hundred figures" spreads out like a heaven of sun, moon, and stars. Verily do we 'behold the model of the world here!' No wonder the translator "prides himself on this production." Of all his numerous works this is his master-piece, or, as he would say, his *chef d'œuvre.* It is his "last and latest" offspring; and as with 'Time' so with Townsend, his 'noblest offspring is the last.' It is the keystone to the arch which spans Lethe for him; the capital to the column on which the light of the world's smile shall ever rest; the last block in the pyramid where he, like Mott, is to lie in "dazzling, enduring, and endearin gbalm" *per sæcula sæculorum!* With a "conscious conviction" of all this, he throws down his pen exclaiming, like Ovid:

'Quàque patet domitis [lingua anglicana] terris,
Ore legar populi: perque omnia sæcula famâ
(Si quid habent veri [mea] presagia) vivam.'

Why not? Is not this translation the "great store-house" of all knowledge, past, present, and to come? Does it not contain "the highly interesting and invaluable details of surgical principles and discoveries, and surgical anatomy and relations, traced down with astonishing erudition and acute discrimination, by the learned author M. Velpeau, from the earliest epochs to the present day;" also "the true history of every operative surgical process, principle or discovery, which adorn[s] or can illustrate the annals of the science, fortified as the whole also is by direct and specific references to every name, authority, or work, ancient or modern, upon which the proofs of his assertions and citations rest?" Is it not "an inexhaustible arsenal or armory for almost every thing that is truly valuable or worthy of preservation," especially of "new matter superadded," "obtained [by the translator] at the cost of much time, and severe researches into, and a close analysis of, all that has been published in different parts of the earth?" And lastly, does it not embrace [that "precious treasure for all future time,"] "the details of the great and master operations, and discoveries, and improvements in surgical processes and principles" of his "long-cherished, personal and honored friend, preceptor and kinsman," [Dr. Mott,] "all now for the first time" "completely arranged and revised in chronological order," "successively as they emanated from his [Dr. Mott's] great practical mind, or were created into existence by his incomparable genius and consummate skill?" "together with his last and latest opinions on these matters," "booked up to the latest moment?" Why, this "first American edition" is a new Pandora's box, filled with every 'good and perfect gift'—a literary ark, from which the deluged earth might be replenished. It is the beginning of a new era in the history of the world!—the era of Velpeau, Townsend, and Mott. It is an epoch in the flight of time—a new starting point, from which time itself must be computed. Almanacs (serious as well as comic) must be changed; calendars must conform to it. We must count hereafter not *Anno Mundi* like Freemasons, nor *Anno Urbis Conditæ* like Romans, nor *Anno Domini* like Christians, nor in the year of the *Hegira* like Mahommedans, nor in the year of the Republic like Frenchmen whilom did; but *Anno Libri Conversi!*

Such being the many-sided and remarkable character of these volumes, it will create no surprise in any one to learn that the translator is completely taken with his own image as reflected in them. It is, as may well be conjectured, of gigantic proportions, vast as the images seen upon the Brocken; and while to our untutored eyes it is a 'perfect fright,' it is to his a 'perfect love.' No beauty *dèbutante*, in full dress for her first *soirèe*, ever half so much admired her pretty face as given back by her Psyche mirror. He is the true Narcissus of authors, translators, and annotators. As Narcissus, like a good-for-nothing scamp, fled through the forest when the gentle Echo wooed him; so Townsend turns his back on Physic, panting for his embrace. Narcissus, after his hasty and ungallant retreat, stooped to quench his thirst, and while drinking, fell in love with his own image in the water; so Townsend, having snubbed Physic, while pouring an inky flood of "superadded new matter" through the pages of Velpeau, straightway falls in love with *his* image.

> 'Dumque [*scribit*], visæ correptus imagine formæ,

> Rem sine corpore amat: corpus putat esse, quod umbra est!'

How often did Narcissus kiss the deceitful waves! and his modern counterpart,

> 'Irrita fallaci quoties dedit oscula [*libro!*]'

(Ghost of the gentle Ovid—of Ovidius *Naso!* turn not up thy handsome nose at our oft interpolations!) But here the parallel must stop, for none but Townsend's 'self *can* be his parallel.' Narcissus pined and died, and was converted into a flower. But Townsend will never die! he is mortal, but immortal too! and when he leaves this earth, it will be by *translation*.

Fixing our mordacious grasp again on the third volume, we observe that the preface is mainly occupied by "reclamations." The reader will already have perceived that this remarkable translation forestalls posterity to some extent, that it pretends to sing of all the immortals 'in their order due and fit degrees,' but especially of all the American surgeons that "occupy an honorable rank in the profession." Accordingly, as a part of this scheme, two pages are occupied in the first volume in glorifying Dr. John W. Schmidt as the first man to divide the masseter muscle. A great fuss was made over this. In the second volume, the translator corrects himself, and devotes four pages and a half to the glorification of Dr. John Murray Carnochan as the *real* "first man" to divide the masseter muscle. This correction he makes with a great deal of alacrity and *empressement.* So fearful indeed was he of doing temporary injustice to Dr. Carnochan, that he did not wait until the second volume was through the press, but published the reclamation at once in the New York Journal of Medicine. But the ghost would not stay laid—the everlasting masseter muscle comes up again in the preface to this "third and last" volume, and this time it evidently *gravels* the translator a good deal. Dieulafoy, of Toulouse, now seems to be the man on whom the burden of this honor lies, and before whom Carnochan must 'pale his ineffectual fires.' The translator fervently hopes that this ghost is "now put forever at rest," and so do we.

Again, it was very strongly impressed on us, that M. V. Mott was absolutely "the first man *in America*" who removed the leg at the thigh-joint! This was a most wonderful feat indeed; but it turns out that it belongs to a Kentucky surgeon, Dr. Brashear, who formerly lived at Bardstown. In this case the translator informs us that "Dr. Mott cheerfully abstracts the plume from his own honored brows that can spare many and much more like this, and affixes it upon the name of the gentleman where it rightly belongs." Magnanimous Mott!

Again: In the first volume the translator takes "occasion to bestow some laudation on Dr. Mott," as the first man to institute "a medical and surgical clinique" in this country. But, small as this matter is, Dr. March, of Albany, puts in his claim; and now mark the sequel. Our translator is "ready to concede to him all the praise he is thereby entitled to;" but, he adds, "the matter of cliniques is quite an old affair all over the world, having been in common vogue for a century, or for half a century at least!" Is not that deliciously cool? Is it not better than the old fable showing how circumstances alter cases? And Dr. Mott, too, with as many plumes on his brow as a Winnebago chief!

And now we are going to put in *our* "reclamation." The translator and his sponsor both seem remarkably fond of the terms "medical university" and "university in medicine." We claim the honor of this "discovery" for the steam doctors of Alabama, and the Solons of that State who "first" gave the steam doctors a charter for the "first" "medical university." We do this "to vindicate the silent and unprotected dead," and to "rebuke the ambitious and reckless living," "a class of persons" "constantlv addicted to the pernicious habit (which to them seems a source of morbid pleasure) of decorating themselves with the plumes which righteously belong to the reputation of others." We hope that "in the next edition" MM. Townsend and Mott will cheerfully abstract this plume from their own honored brows, that can spare *many and much more like this*, and affix it where it belongs. Or will they, like Falstaff, find some 'trick,' some 'device,' some 'starting-hole to hide them from this open and apparent shame?'

But here, for the present, we must leave this "great work." The holidays are upon us, and we are scarce equal to them and Townsend together. Besides, it is unphilosophical for one to crowd all his enjoyments into the very shortest space of time. We shall therefore make another paper, and then our notices will correspond in number with these volumes. It is possible, too, that we shall wind up with a "concluding appendix" in which "justice" will be done to every body.

